# The efficacy of nisin against *Listeria monocytogenes* on cold-smoked salmon at natural contamination levels is concentration-dependent and varies by serotype

**DOI:** 10.3389/fmicb.2022.930400

**Published:** 2022-09-06

**Authors:** Ruixi Chen, Jordan William Skeens, Martin Wiedmann, Veronica Guariglia-Oropeza

**Affiliations:** Department of Food Science, Cornell University, Ithaca, NY, United States

**Keywords:** *Listeria monocytogenes*, nisin, cold-smoked salmon, antimicrobial concentration, serotype

## Abstract

Cold-smoked salmon is a ready-to-eat food product capable of supporting *Listeria monocytogenes* growth at refrigeration temperatures. While the FDA-approved antimicrobial nisin can be used to mitigate *L. monocytogenes* contamination, stresses associated with cold-smoked salmon and the associated processing environments may reduce nisin efficacy. A previous study in our laboratory showed that, at high inoculation levels, pre-exposure of *L. monocytogenes* to sublethal concentrations of quaternary ammonium compounds had an overall detrimental effect on nisin efficacy. The objective of this study was to investigate the impact of nisin concentration and storage temperature on nisin efficacy against *L. monocytogenes* inoculated on salmon at natural contamination levels. Three *L. monocytogenes* strains were pre-grown in the presence of sublethal levels of benzalkonium chloride prior to inoculation at ~10^2^ CFU/g on salmon slices that were pre-treated with either 0, 25, or 250 ppm nisin, followed by vacuum-packing and incubation at 4 or 7°C for up to 30 days. *L. monocytogenes* was enumerated on days 1, 15, and 30 using direct plating and/or most probable number methods. A hurdle model was constructed to describe the odds of complete elimination of *L. monocytogenes* on salmon and the level of *L. monocytogenes* when complete elimination was not achieved. Our data showed that (i) nisin efficacy (defined as *L. monocytogenes* reduction relative to the untreated control) was concentration-dependent with increased efficacy at 250 ppm nisin, and that (ii) 250 ppm nisin treatments led to a reduction in *L. monocytogenes* prevalence, independent of storage temperature and serotype; this effect of nisin could only be identified since low inoculation levels were used. While lower storage temperatures (i.e., 4°C) yielded lowered absolute *L. monocytogenes* counts on days 15 and 30 (as compared to 7°C), nisin efficacy did not differ between these two temperatures. Finally, the serotype 1/2b strain was found to be more susceptible to nisin compared with serotype 1/2a and 4b strains on samples incubated at 7°C or treated with 25 ppm nisin. This variation of nisin susceptibility across serotypes, which is affected by both the storage temperature and nisin concentration, needs to be considered while evaluating the efficacy of nisin.

## Introduction

*Listeria* (*L.*) *monocytogenes* is a Gram-positive human pathogen, which can cause listeriosis, a potentially life-threatening disease that primarily affects pregnant women (who can pass it on to their newborns), the elderly, and immunocompromised individuals (Farber and Peterkin, 1991; Center for Disease Control, 2020). Since 99% of the human listeriosis cases in the US can be attributed to consumption of contaminated foods (Scallan et al., 2011), controlling *L. monocytogenes* in foods is of crucial importance. However, prevention of *L. monocytogenes* contamination remains extremely challenging due to the wide distribution of *L. monocytogenes* in natural as well as urban environments (Nightingale et al., 2004; Sauders et al., 2012), food processing facilities (Møretrø and Langsrud, 2004; Ferreira et al., 2014), and consumer homes (Ding et al., 2013).

*Listeria monocytogenes* contamination represents a particular concern and economic burden for the cold-smoked salmon industry. *L. monocytogenes* has been reported to be frequently found in raw materials (i.e., salmon; Eklund et al., 1995; Di Ciccio et al., 2012) and smoked seafood processing environments (Dauphin et al., 2001; Vogel et al., 2001; Di Ciccio et al., 2012; Nakari et al., 2014). Importantly, the process of cold smoking (usually at a temperature less than 30°C) does not represent an effective kill step for *L. monocytogenes* (Eklund et al., 1995; Cornu et al., 2006). Although the initial *L. monocytogenes* contamination levels are usually low (Rørvik, 2000), samples with *L. monocytogenes* levels exceeding 10^6^ CFU/g have been reported (Acciari et al., 2017), as the time and temperature of storage (Kang et al., 2012) as well as the intrinsic characteristics of smoked fish (e.g., water activity, pH, and salt content) normally fall within a range that allows for growth of *L. monocytogenes* (Seeliger and Jones, 1984). Consequently, cold-smoked salmon products have been associated with a number of listeriosis outbreaks and food recalls worldwide (Goetz, 2013; Nakari et al., 2014; Gillesberg Lassen et al., 2016; Schjørring et al., 2017; Vincent and Merchant, 2018).

One possible strategy to control *L. monocytogenes* contamination of cold-smoked salmon products is the application of FDA-approved, Generally Recognized As Safe (GRAS) bacteriocins (Mokoena, 2017). Nisin, a bacteriocin naturally produced by some strains of *Lactococcus lactis*, exhibits antimicrobial activity against a broad range of Gram-positive spoilage microorganisms and foodborne pathogens, including *L. monocytogenes* (Singh, 2018). Nisin kills bacterial cells mainly by recognizing and binding to lipid II in cell membrane, which is used as a “docking molecule” to assemble and form pores efficiently, leading to dissipation of the proton motive force (Brötz et al., 1998; Breukink et al., 1999; Breukink and de Kruijff, 2006).

Commercialized nisin has been used for controlling *L. monocytogenes* on cold-smoked salmon products (Neetoo and Mahomoodally, 2014), and while a specific limit has not been stipulated for cold-smoked salmon, the maximum limit of nisin in pasteurized processed cheese spreads has been set to 10,000 IU/g (250 ppm) by FDA (Cleveland et al., 2001). The efficacy of nisin treatments against *L. monocytogenes* on cold-smoked salmon, either by itself or in synergy with other treatments, has been extensively studied at various antimicrobial concentrations and storage temperatures. Several studies were consistent in showing that when challenged with up to 50 ppm nisin on cold-smoked salmon, *L. monocytogenes* experienced an initial decrease in level, followed by a potential regrowth of the population to higher levels (Tang et al., 2013; Kang et al., 2014; Chen et al., 2020). Furthermore, the susceptibility of *L. monocytogenes* to nisin treatments has been reported to be affected by pre-growth condition in growth media (Kang et al., 2015) and by both pre-growth condition and strain diversity on cold-smoked salmon (Chen et al., 2020). Consistent with this, incorporating multiple strains and pre-growth conditions in challenge studies associated with foodborne pathogens has also been suggested by Harrand et al. (2019) and the EURL *Lm* Technical Guidance Document on challenge tests and durability studies for assessing shelf-life of ready-to-eat foods related to *L. monocytogenes* (Bergis et al., 2021). Notably, the aforementioned challenge studies (Tang et al., 2013; Kang et al., 2014; Chen et al., 2020) on cold-smoked salmon were conducted at 7°C (mimicking a slight temperature abuse at the consumer phase) and with high inoculum levels (10^4^–10^6^ CFU/g). Since storage temperature and inoculum level have been reported to impact the apparent efficacy of nisin treatments (Neetoo et al., 2008), it is essential to validate efficacy of nisin treatments against *L. monocytogenes* on cold-smoked salmon at natural contamination levels and lower temperatures. Moreover, the efficacy of nisin treatments at concentrations higher than 50 ppm for controlling *L. monocytogenes* on cold-smoked salmon remains to be explored.

The objectives of this study were to (i) validate nisin efficacy for controlling *L. monocytogenes* on cold-smoked salmon at low contamination levels (i.e., 10^2^ CFU/g) that are more reflective of natural contamination (European Food Safety Authority, 2013, 2014), as the use of artificially high contamination levels (e.g., 10^6^ CFU/g) may not be reflective of the real-world industries and may sometimes overestimate the efficacy of antimicrobial treatments (Yoon et al., 2005; NACMCF, 2010; Spanu et al., 2014), and (ii) explore the impact of antimicrobial concentration and storage temperature on nisin efficacy, under a worst-case scenario (i.e., pre-exposure to sublethal concentrations of quaternary ammonium compounds, which has been shown to decrease nisin susceptibility) that is universally applicable to different *L. monocytogenes* strains (Chen et al., 2020).

## Materials and methods

### Bacterial strains and culture preparation

Three *L. monocytogenes* strains (Table 1) were selected for this study because they (i) represented the serotypes (i.e., 1/2a, 1/2b, and 4b) commonly associated with human listeriosis and cold-smoked salmon production (Farber and Peterkin, 1991; Clark et al., 2010; Di Ciccio et al., 2012; Vongkamjan et al., 2013), (ii) were isolated from smoked fish finished products, and (iii) were reported to show high, medium, and low nisin susceptibility in previous challenge studies for *L. monocytogenes* on cold-smoked salmon (Tang et al., 2013; Kang et al., 2014; Chen et al., 2020). These strains were preserved in Brain Heart Infusion (BHI) broth with 15% (v/v) glycerol at −80°C. Prior to experiments on cold-smoked salmon, frozen stock cultures were streaked on BHI agar plates, followed by incubation at 37°C for 20–24 h. Inoculum preparation was performed as previously described (Chen et al., 2020) to simulate pre-exposure of *L. monocytogenes* to sublethal concentrations of quaternary ammonium compounds; this pre-exposure was selected as a worst-case scenario as it has been reported (Chen et al., 2020) to reduce nisin efficacy. Briefly, for each strain, single colonies from freshly streaked plates (less than 7 days old) were inoculated into 5 ml BHI broth, followed by incubation with shaking (200 rpm) at 37°C for 18 h. Bacterial cultures were then sub-cultured (1:100) into BHI broth with benzalkonium chloride (final concentration: 0.5 μg/ml) and subsequently incubated at 7°C until mid-logarithmic phase, as previously detailed (Chen et al., 2020).

**Table 1:** *Listeria monocytogenes* strains used in this study.

FSL number^a^	Lineage	Serotype	Ribotype	Source of isolation	Year	References
FSL F2-0237	II	1/2a	DUP-1062D	Finished RTE food product (salmon)	1999	Sauders et al., 2012
FSL L3-0051	I	1/2b	DUP-1042C	Finished RTE food product (salmon)	2002	Sauders et al., 2012
FSL F2-0310	I	4b	DUP-1038B	Finished RTE food product (salmon)	2000	Sauders et al., 2012

a Food Safety Lab (FSL) strain information can be found on Food Microbe Tracker, available at: http://www.foodmicrobetracker.com/.

### Nisin stock solution preparation

A commercial preparation of nisin (Nisaplin, containing 2.5% nisin) was provided by DuPont (DuPont, Wilmington, DE). Immediately prior to each experiment, Nisaplin powder (500 mg or 5,000 mg) was added to ultrapure water (15 ml) in a 50 ml centrifuge tube, followed by vortexing until complete dissolution. The nisin concentrations of the stock solution were approximately 833.3 and 8333.3 ppm, which corresponded to a final concentration of 25 and 250 ppm on salmon, respectively, when adding 300 μl of the stock solution onto a 10 ± 0.5 g salmon slice.

### Cold-smoked salmon sample preparation

Pre-sliced, vacuum-packed cold-smoked salmon containing celery extract as the nitrite source was provided by Acme Smoked Fish Corporation. A single batch of salmon (300 g per package) was shipped frozen to our laboratory and stored at −20°C. Prior to each experiment, one package was thawed at 4°C overnight. For each *L. monocytogenes* strain, a bacterial culture was prepared as described in section 2.1, and salmon inoculation was performed as previously reported (Kang et al., 2014; Chen et al., 2020). Briefly, for each experiment, 10 ± 0.5 g salmon slices were prepared aseptically for inoculation with each of the three *L. monocytogenes* strains as well as a uninoculated control, two treatments (untreated and treated with either 25 or 250 ppm nisin), and three sampling days during storage (days 1, 15, and 30) for a total of 24 samples. For each of the nisin-treated samples, 300 μl nisin stock solution prepared as described in section 2.2 was evenly distributed on the surface of the salmon slice and further spread with a sterile spreader. The samples were incubated in a biosafety cabinet (NuAire, Inc., Plymouth, MN) for 30 min to facilitate complete absorption of nisin to the surface of salmon. For each *L. monocytogenes* strain, the OD_600_ value of bacterial culture was measured to confirm that mid-logarithmic growth phase was reached. The bacterial culture was subsequently diluted and inoculated onto the surface of salmon slices at a final concentration of ~10^2^ CFU/g. The inoculated samples were incubated in a biosafety cabinet for another 30 min, vacuum-packed in Whirl-Pak^®^ filter bags (0.33 mm pore size; oxygen transmission rate: 149.9 cc/in^2^/24 h; North American Sales Company, Inc., Pacific Palisades, CA), and incubated at 4 or 7°C for up to 30 days. Three biological replicates were conducted for each combination of nisin concentration (25 ppm vs. 250 ppm) and storage temperature (4°C vs. 7°C).

### Evaluation of nisin efficacy against *Listeria monocytogenes* on cold-smoked salmon

For each experiment, *L. monocytogenes* enumeration was performed on days 1, 15, and 30 of incubation, using direct plating on selective and differential media and/or the most probable number (MPN) technique. MPN was used for enumerating *L. monocytogenes* numbers on samples processed on day 1 due to the low inoculation level used. On days 15 and 30, *L. monocytogenes* levels were estimated based on the data obtained from previous experiments; one or both enumeration method(s) were used for each sample such that the range of measurement would most likely cover the actual level of *L. monocytogenes*. Both enumeration methods (MPN and direct plating) are further described in the following sections. Uninoculated samples (negative controls) were processed on each day of enumeration to monitor natural contamination of salmon samples with *L. monocytogenes*, following the FDA bacteriological analytical manual (BAM) procedures for enrichment and isolation of *L. monocytogenes* in food (US Food and Drug Administration, 2017). None of the negative controls in this study yielded colonies with typical *Listeria* morphology, suggesting (i) no natural and cross contamination of the samples with *L. monocytogenes* and (ii) the salmon native microbiota did not contain organisms that share similar morphologies as *L. monocytogenes* on the selective and differential media used.

#### Quantification by MPN

MPN was performed as described in BAM with modifications (US Food and Drug Administration, 2017). Briefly, each sample was diluted with 90 ml of Buffered *Listeria* Enrichment Broth (BLEB) containing selective agents (acriflavine: 10 mg/l; cycloheximide: 40 mg/l; and sodium nalidixic acid: 50 mg/l) and stomached in the filter bag for 1 min at 260 rpm using a Seward stomacher 400 circulator (Seward Limited, Worthing, United Kingdom). A 4-dilution, 3-tube MPN was then prepared in BLEB containing selective agents using the salmon homogenate (obtained from the clean side of the filter bags used); the 4 dilutions were set up to represent appropriate 10-fold dilutions of the salmon homogenate. The MPN enrichment aliquots were incubated at 30°C for 48 h, along with the rest of the salmon homogenate to achieve an overall detection limit of 0.1 MPN/g. Following incubation, samples were streaked (20 μl) onto Modified Oxford Agar (MOX) plates in duplicate to determine the presence/absence of typical *L. monocytogenes* colonies. All MOX plates were incubated at 30°C for 48 h. For each sample, aliquots that tested positive for *L. monocytogenes* were recorded, followed by calculation of *L. monocytogenes* levels (MPN/g) using the MPN v 0.3.0 package (Ferguson and Ihrie, 2019) in R Statistical Programming Environment (R) v 3.5.2 (R Core Team, 2020).

#### Quantification by direct plating

For direct plating, each sample was diluted (1:10) and stomached in the filter bag for 1 min at 260 rpm. Subsequently, salmon homogenate was serially diluted with 1% peptone water and appropriate dilutions were spread-plated onto MOX plates in duplicate. All MOX plates were incubated at 30°C for 48 h, followed by enumeration of typical *L. monocytogenes* colonies using a Sphereflash^®^ Automated Colony Counter (Neutec, Albuquerque, NM) to determine *L. monocytogenes* levels (CFU/g).

### Statistical analysis

All statistical analyses were performed in R (R Core Team, 2020); raw data and R codes are available on Github.^1^ The threshold of significance for all statistical tests was set to *p* = 0.05. Raw *L. monocytogenes* enumeration data estimated using direct plating (CFU/g) and MPN (MPN/g) were decimal log transformed into log_10_(CFU/g) and log_10_(MPN/g), respectively. Due to the superior performance of MPN in estimating viable number of bacterial cells at low concentrations, the log_10_(MPN/g) estimate was primarily used to represent *L. monocytogenes* levels on salmon samples. For samples where log_10_(MPN/g) estimates were not available or beyond the quantifiable range, log_10_(CFU/g) was converted to log_10_(MPN/g) using equation (1), which was generated by fitting a simple linear regression model to preliminary enumeration data of *L. monocytogenes* on samples analyzed by both enumeration methods (Supplementary Figure 1).Consistent with a number of previous studies, the reduction in log_10_(MPN/g) between untreated and nisin-treated samples (hereafter referred to as “log reduction”) was used to infer the overall efficacy of nisin treatments to reduce *L. monocytogenes* levels on salmon; this log reduction could be a result of (i) reductions of *L. monocytogenes* prevalence/levels during the initial killing phase and/or (ii) reduced *L. monocytogenes* growth in the following regrowth phase.


(1) $$\log_{10}\left(MPN/g\right)=1.007\times \log_{10}\left(CFU/g\right)+0.035$$

It was assumed that *L. monocytogenes* levels on nisin-treated samples were governed by two distinct processes; one process determined whether *L. monocytogenes* was present or not, while the other process determined the distribution of detectable counts of *L. monocytogenes*. To be able to investigate both processes, a hurdle model was constructed, which comprised (i) mixed effects logistic models for describing the odds of complete elimination of *L. monocytogenes* due to 250 ppm nisin treatments and (ii) a mixed effects linear model for describing *L. monocytogenes* levels when complete elimination was not achieved. As all inoculated samples that tested negative for *L. monocytogenes* were treated with 250 ppm nisin, the Chi-Square test of independence was first performed to assess the association between the complete elimination of *L. monocytogenes* (i.e., absence in the complete 10 g sample) and 250 ppm nisin treatments. To investigate the odds of the complete elimination of *L. monocytogenes*, 250 ppm nisin-treated samples inoculated with the same strain and stored at the same temperature, regardless of storage days, were treated as replicates. The data of 250 ppm nisin-treated samples were fitted with two mixed effects logistic models (the Serotype Elimination Model and the Temperature Elimination Model) using the lme4 v 1.1.21 package (Bates et al., 2015) to assess the impact of serotype and storage temperature on the odds of complete elimination, respectively. For both models, the outcome specified whether *L. monocytogenes* was completely eliminated, and the random effect was the “age” (i.e., duration of frozen storage at −20°C prior to experiment) of the salmon samples. The fixed effect was serotype (reference level: 1/2a) for the Serotype Elimination Model and storage temperature (reference level: 4°C) for the Temperature Elimination Model. For each model, adding additional variables or interactions did not significantly improve the performance according to the likelihood ratio test. For the primary variable of interest (i.e., the fixed effect) of each model, the odds ratio as well as the 95% confidence interval (CI) were estimated, using the broom.mixed v 0.2.6 package (Bolker and Robinson, 2020) for each alternative level in comparison with the reference level. In addition, a mixed effects logistic model specifying storage day as the fixed effect (reference level: day 1) was also constructed (the Day Elimination Model), and the odds ratios and CIs were estimated as part of the justification for treating samples from different storage days as replicates. A *post hoc* sample size calculation was performed to determine the number of 250 ppm nisin-treated samples necessary for obtaining significant odds ratios.

Due to the reduction in *L. monocytogenes* prevalence among 250 ppm nisin-treated samples, to estimate the theoretical initial log reduction that can be achieved, the following equation described by Pouillot et al. (2015) was used.


(2) $$P_{\mathrm{new}}=P_{\mathrm{original}}\left({\left(1–10^{-D}\right)}^{N_{0}}\right)$$

where *P_original_* and *P_new_* are the prevalence of *L. monocytogenes* among salmon samples before and after the nisin treatment, respectively, *N_0_* is the number of *L. monocytogenes* on samples prior to the nisin treatment, and *D* is the theoretical reduction in log_10_(MPN/g) that can be achieved by nisin treatments.

To investigate the effect of different factors on *L. monocytogenes* levels on cold-smoked salmon when complete elimination was not achieved, the data for samples with detectable levels of *L. monocytogenes* were fitted with a mixed effects linear model (the Level Model). The outcome of the model was the log_10_(MPN/g) of *L. monocytogenes* on the samples. Fixed effects included in the model were: (i) nisin concentration, (ii) storage temperature, (iii) serotype, and (iv) day in storage; two-way interactions included were those between (i) nisin concentration and serotype, (ii) nisin concentration and day in storage, (iii) storage temperature and serotype, and (iv) storage temperature and day in storage. All fixed effects were considered as primary variables of interest, and a backwards stepwise selection was performed to determine the interactions to be retained using (i) the F test and (ii) the likelihood ratio test. The “age” of the salmon samples was included in the model as a random effect. A two-way ANOVA was performed on the model to evaluate the impact of the main effects as well as the interactions on the outcome. *Post hoc* pairwise comparison (i.e., Tukey’s HSD test) was performed, using the emmeans v 1.4.4 package (Lenth, 2020), for the main effects and interactions that significantly affected the outcome.

## Results

### The effect of 250 ppm nisin against *Listeria monocytogenes* on cold-smoked salmon involves a stochastic process of complete elimination, which was not affected by storage temperature or serotype

**Figure 1:**
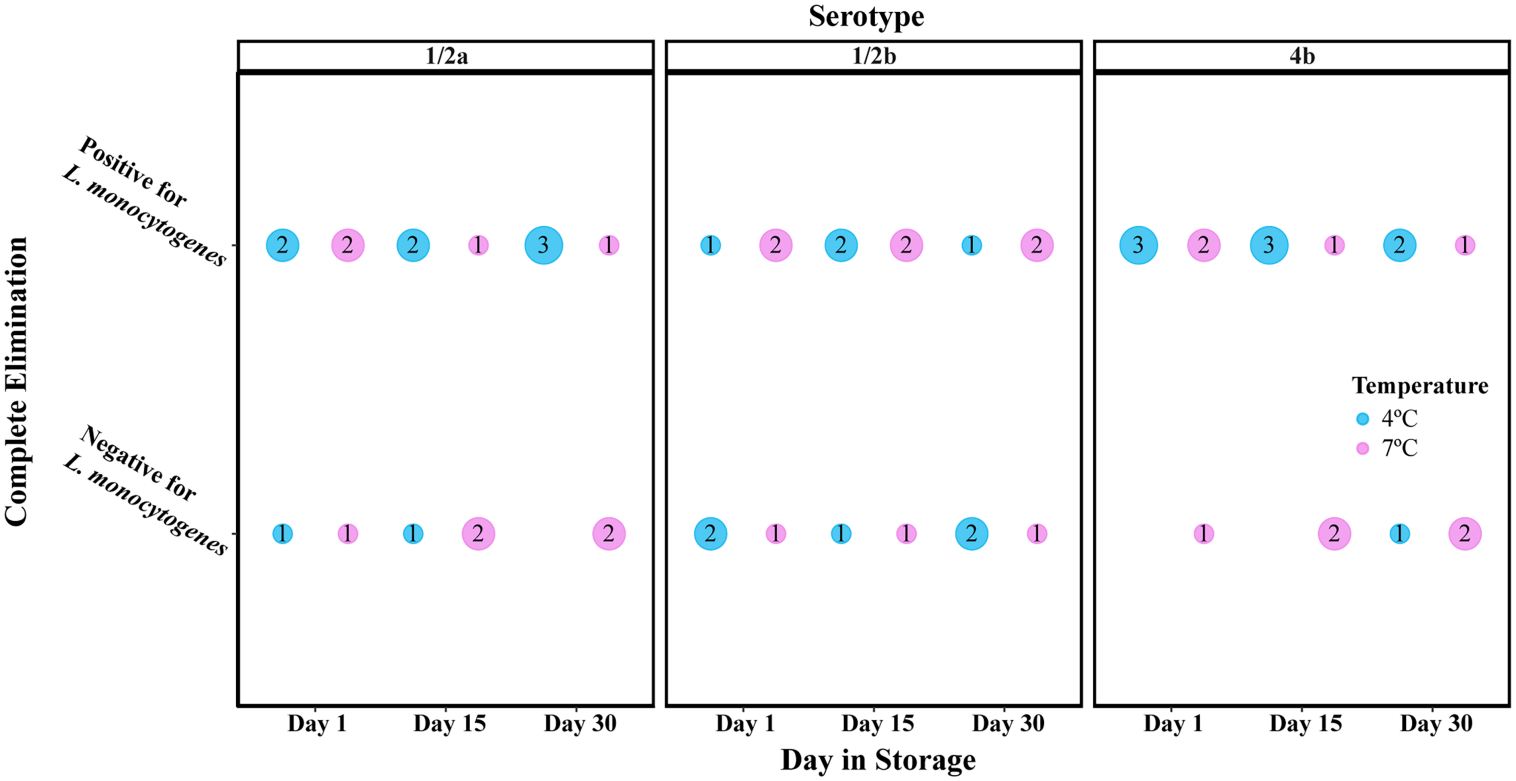
Presence/absence of *Listeria monocytogenes* on 10-g cold-smoked salmon samples treated with 250 ppm nisin. For each combination of storage temperature, serotype, and day in storage, the number of samples positive and negative for *L. monocytogenes* are indicated in the center of the circles. The size of the circles is in proportion to the number of samples.

Treatment of cold-smoked salmon with 250 ppm nisin resulted in one of two scenarios: (i) complete elimination of *L. monocytogenes* or (ii) incomplete elimination, recovery, and growth of *L. monocytogenes*. Untreated or 25 ppm nisin-treated samples, on the other hand, all tested positive for *L. monocytogenes*. A Chi-Square test of independence indicated a significant association (*p* < 0.001) between the complete elimination and the 250 ppm nisin treatments. To investigate the odds of complete elimination due to 250 ppm nisin treatment, we assumed that this event occurred within the first 24 h post-inoculation based on two pieces of evidence. Firstly, growth curves of *L. monocytogenes* on 50 ppm nisin-treated salmon retrieved from Tang et al. (2013) suggested nisin was most efficient in killing *L. monocytogenes* at or around 0.44–2.25 days post-inoculation. Since the current study involved a lower inoculum size (10^2^ CFU/g instead of 10^4^ CFU/g) and a much higher nisin concentration (250 ppm instead of 50 ppm) compared with Tang et al. (2013), it is likely that the complete elimination would be achieved within the first 24 h. Secondly, according to the Day Elimination Model, the odds of complete elimination on days 15 and 30 were not significantly different compared to day 1 (Table 2). Therefore, 250 ppm nisin-treated samples inoculated with a given *L. monocytogenes* strain and stored at the same temperature were considered as replicates for calculating the odds of complete elimination. At a lower detection limit of 0.1 MPN/g, *L. monocytogenes* was undetectable on 21 (39%) of the 54 samples, indicating the potential of highly concentrated nisin treatments to reduce the prevalence of *L. monocytogenes* contamination. For a given combination of serotype and storage temperature, the proportion of samples that showed complete *L. monocytogenes* elimination ranged from 1/9 for serotype 4b at 4°C to 5/9 for serotype 1/2b at 4°C and serotype 1/2a as well as 4b at 7°C (Figure 1). The Temperature Elimination Model estimated an odds ratio of 2.21 (95% CI: 0.72, 6.75; *p* = 0.166) for 7°C in comparison with 4°C, and the Serotype Elimination Model estimated odds ratios of 1.26 (95% CI: 0.33, 4.74; *p* = 0.735) and 0.79 (95% CI: 0.20, 3.07; *p* = 0.729) for serotype 1/2b and 4b, respectively, in comparison with serotype 1/2a (Table 2), indicating that the odds of complete elimination of *L. monocytogenes* on cold-smoked salmon were not significantly affected by strain serotype and storage temperature.

**Table 2:** Odds ratios and 95% confidence intervals (CIs) with respect to factors associated with the complete elimination of *Listeria monocytogenes* on cold-smoked salmon samples due to 250 ppm nisin treatments.

Factors (levels)^a^	Odds ratio	95% confidence interval	*p* value
**Serotype**			
1/2a	1.00	–	–
1/2b	1.26	0.33, 4.74	0.735
4b	0.79	0.20, 3.07	0.729
**Storage temperature**			
4°C	1.00	–	–
7°C	2.21	0.72, 6.75	0.166
**Storage day**			
1	1.00	–	–
15	1.27	0.33, 4.97	0.729
30	1.60	0.41, 6.18	0.495

a The impact of each factor on the complete elimination was assessed using a mixed effects logistic model at the univariable level. One of the levels was selected as the reference level for calculating odds ratios and CIs.

### Nisin reduces the level of *Listeria monocytogenes* on cold-smoked salmon in a concentration-dependent manner throughout the storage

**Figure 2:**
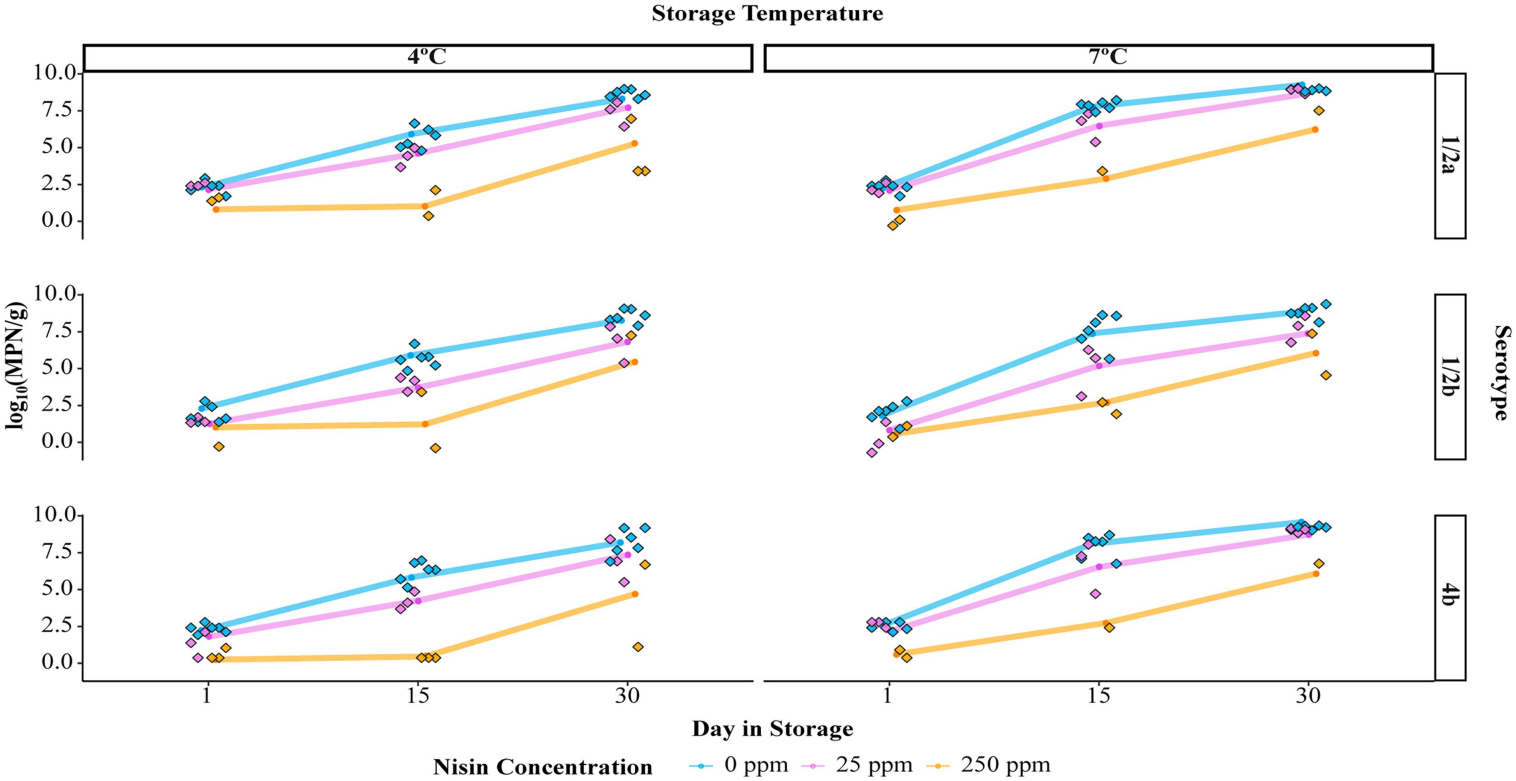
Levels of *Listeria monocytogenes* [in log_10_(MPN/g)] on cold-smoked salmon treated with different concentrations of nisin and stored for up to 30 days. The results are displayed by storage temperature (4°C or 7°C) and serotype of *L. monocytogenes* (1/2a, 1/2b, or 4b). Raw data points are plotted as standalone diamonds and estimated marginal means of the *L. monocytogenes* level (EMMLM) obtained from the mixed effects linear model (the Level Model) are displayed as colored dots connected by solid lines.

In 25 ppm nisin-treated samples and 250 ppm nisin-treated samples in which complete elimination was not achieved, a nisin concentration-dependent reduction in *L. monocytogenes* numbers was observed throughout the storage (Figure 2 and Supplementary Table 1). Specifically, the log_10_(MPN/g) of *L. monocytogenes* on 0, 25, and 250 ppm nisin-treated samples, averaged over serotypes and temperatures, was 6.82, 5.13, and 1.55 on day 15 and 8.74, 7.78, and 5.50 on day 30. Compared to days 15 and 30, however, the efficacy of nisin treatments for reducing *L. monocytogenes* levels was less pronounced on day 1, as the log_10_(MPN/g) was only reduced from 2.24 in samples with 0 ppm nisin to 1.72 and 0.59 in samples with 25 and 250 ppm nisin, respectively. In addition, leveraging the *L. monocytogenes* prevalence reduction (from 100 to 61%) among samples treated with 250 ppm nisin, the theoretical initial log reduction due to 250 ppm nisin treatments was estimated to be 3.025. Altogether, these results suggest that 250 ppm nisin treatments have a higher efficacy, compared to 25 ppm nisin treatments, in reducing *L. monocytogenes* levels on cold-smoked salmon.

**Figure 3:**
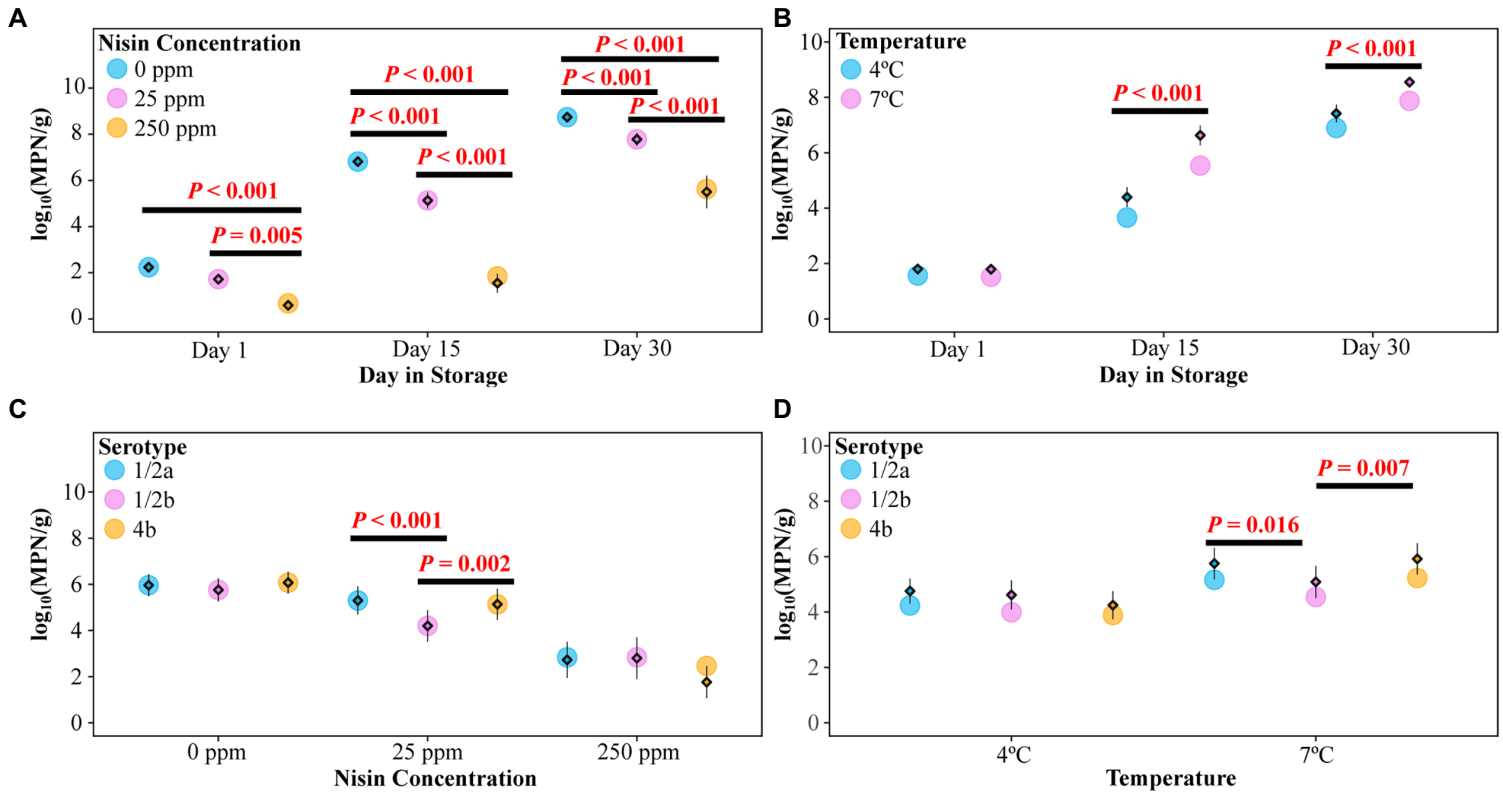
*Listeria monocytogenes* levels [in log_10_(MPN/g)] on cold-smoked salmon treated with different nisin concentrations and tested on days 1, 15, and 30. Within each panel, raw data points are summarized as means (standalone diamonds) ± standard errors (shown as error bars, which sometimes are not visible due to their small values, e.g., the day 1 data shown in panel A) and estimated marginal means of the *L. monocytogenes* level (EMMLM) obtained from the mixed effects linear model (the Level Model) were plotted as circles. **(A)** EMMLMs (averaged over serotypes and storage temperatures) compared across different nisin concentrations on each day in storage. **(B)** EMMLMs (averaged over nisin concentrations and serotypes) compared between storage temperatures on each day in storage. **(C)** EMMLMs (averaged over storage temperatures and days in storage) compared across different serotypes for each nisin concentration. **(D)** EMMLMs (averaged over nisin concentrations and days in storage) compared across different serotypes for each storage temperature. Pairwise comparison of the EMMLMs (within each category shown on the X axis of each panel) was performed using the Tukey’s HSD test (overall *α* = 0.05). Significant differences were indicated by the *p* values above horizontal lines.

A mixed effects linear model (the Level Model) was developed to describe the effect of nisin concentration, serotype, storage temperature, day in storage, and four two-way interactions on the levels of *L. monocytogenes* on cold-smoked salmon (see Table 3 for summary statistics of the two-way ANOVA). According to the F tests, all main effects as well as two-way interactions included in the model were significant (*p* < 0.05). However, interpretation was only deemed appropriate for the two-way interactions, which involved each of the main effects. The two-way interaction between nisin concentration and day in storage was determined to be significant (*p* < 0.001), suggesting the impact of nisin concentration on *L. monocytogenes* levels (represented by log_10_(MPN/g)) on salmon varied across different days in storage. A pairwise comparison analysis using Tukey’s HSD test was performed to compare the model-reported estimated marginal means of the *L. monocytogenes* level (hereafter referred to as “EMMLM”), averaged over serotypes and storage temperatures, across different nisin concentrations on each day in storage. The EMMLM for the 250 ppm nisin treatment was significantly lower (adj. *p* < 0.05) compared to 0 and 25 ppm nisin treatments for all days in storage (Figure 3A). Compared to untreated samples, the samples treated with 25 ppm nisin showed a significantly lower EMMLM on days 15 and 30 (adj. *p* < 0.05), with no significant differences observed for day 1 (Figure 3A). These results confirm that the 250 ppm nisin treatment has a higher efficacy in reducing *L. monocytogenes* levels on cold-smoked salmon throughout the storage as compared to the 25 ppm nisin treatment.

**Table 3:** Two-way ANOVA summary of the mixed effects linear model (Level Model).

	Sum sq^a^	Mean sq^a^	NumDF^a^	DenDF^a^	*F* value^a^	Pr(>F)^a^
Nisin concentration	248.58	124.29	2	130.58	186.59	<0.001
Storage temperature	20.38	20.38	1	9.89	30.60	<0.001
Serotype	4.72	2.36	2	169.91	3.54	0.031
Day in storage	869.13	434.57	2	166.69	652.39	<0.001
Nisin concentration: Serotype	8.44	2.11	4	169.03	3.17	0.015
Nisin concentration: Day in storage	50.49	12.62	4	166.46	18.95	<0.001
Storage temperature: Serotype	4.94	2.47	2	167.02	3.71	0.027
Storage temperature: Day in storage	29.94	14.97	2	165.90	22.47	<0.001

a Two-way ANOVA statistics of the mixed effects linear model by reference coding (R default). Sum sq.: the sum of squares due to the factor or two-way interaction; Mean sq.: mean of the sum of squares due to the factor or two-way interaction; NumDF: numerator degree of freedom; DenDF: denominator degree of freedom; *F* value: the F-statistic; Pr(>F): the *p* value.

### Lower storage temperature is associated with reduced *Listeria monocytogenes* level, but does not affect nisin efficacy on cold-smoked salmon

In the Level Model (Table 3), the two-way interaction between storage temperature and day in storage was significant (*p* < 0.001), indicating that the effect of storage temperature on *L. monocytogenes* levels on salmon was dependent on days in storage. Specifically, although no difference in *L. monocytogenes* levels on salmon was observed on day 1, for both days 15 and 30 salmon stored at 4°C showed significantly lower *L. monocytogenes* levels (adj. *p* < 0.001) as compared to salmon stored at 7°C, as indicated by the EMMLM averaged over serotypes and nisin concentrations (Figure 3B). While these data supported more rapid growth of *L. monocytogenes* on salmon stored at 7°C (as compared to 4°C), storage temperature (i.e., 4 or 7°C) did not seem to affect the overall efficacy (i.e., *L. monocytogenes* reduction relative to the untreated controls) of nisin treatment throughout storage, since neither the two-way interaction between nisin concentration and storage temperature nor the three-way interaction between nisin concentration, storage temperature, and day in storage were identified as significant and retained in the Level Model. Importantly, this may suggest that (i) storage at mildly abusive temperature has limited impact on the log reduction achieved during the initial killing phase and (ii) the effect of temperature on *L. monocytogenes* growth does not differ substantially between untreated and nisin-treated samples.

### The effect of serotype on *Listeria monocytogenes* levels on cold-smoked salmon is dependent on nisin concentration and storage temperature

Significant interactions were identified between serotype and nisin concentration (*p* = 0.015) as well as between serotype and storage temperature (*p* = 0.027), indicating that *L. monocytogenes* strains from different serotypes grew to different levels in a nisin concentration and storage temperature dependent manner. Compared with the strains representing serotypes 1/2a (FSL F2-0237) and 4b (FSL F2-0310), the EMMLM was significantly lower for the serotype 1/2b strain (FSL L3-0051) on salmon treated with 25 ppm nisin (averaged over storage temperatures and days in storage) or on salmon stored at 7°C (averaged over nisin concentrations and days in storage; Figures 3C,D, respectively). Overall, this indicates that the 1/2b strain was more susceptible to 25 ppm nisin treatment or grew with a slower rate at 7°C compared to the strains from other serotypes. While the three-way interaction between serotype, nisin concentration, and storage temperature was not found to be significant, the raw data suggested *L. monocytogenes* levels on salmon treated with 25 ppm nisin, averaged over days in storage, were consistently lower for the serotype 1/2b strain [log_10_(MPN/g) = 4.33], compared with the strains from serotypes 1/2a and 4b [log_10_(MPN/g) = 5.86 and 6.12 for the 1/2a and 4b strains, respectively], following incubation at 7°C (Supplementary Table 1). This difference was not as apparent when the samples were stored at 4°C [log_10_(MPN/g) = 4.08, 4.74 and 4.15 for the 1/2b, 1/2a, and 4b strains, respectively]. This suggests that the susceptibility of *L. monocytogenes* to 25 ppm nisin may differ across serotypes at 7°C but to a lesser extent at 4°C. Notably, the observed serotype-dependent nisin susceptibility of *L. monocytogenes* may represent strain differences, since only one strain was included in this study to represent each of the serotypes.

## Discussion

Assessments of nisin efficacy in reducing *L. monocytogenes* levels on cold-smoked salmon have typically been conducted with high inoculation levels, at least in part due to the increased enumeration errors associated with low bacterial concentrations (Spanu et al., 2014). However, it is important to validate the efficacy of nisin treatments at natural contamination levels to better assess their “real world” efficacy. In the current study, the efficacy of nisin treatments to control *L. monocytogenes* on cold-smoked salmon was thus assessed at an inoculum level that resembles natural contamination levels. To reduce enumeration errors associated with low *L. monocytogenes* numbers, we used a combination of MPN and direct plating approaches to achieve an overall lower detection limit of 0.1 MPN/g. Our results suggest that (i) treatments with high levels of nisin can reduce the *L. monocytogenes* prevalence among cold-smoked salmon products; (ii) nisin efficacy against *L. monocytogenes* on cold-smoked salmon does not appear to be reduced by storage at mildly abusive temperature (i.e., 7°C); and (iii) serotype/strain diversity needs to be considered in challenge studies as nisin efficacy varies depending on serotype; the variation in nisin efficacy by serotype is in turn affected by antimicrobial concentration and storage temperature.

### Treatment with high levels of nisin can reduce the *Listeria monocytogenes* prevalence among cold-smoked salmon products

Our data revealed that increasing nisin concentrations from 25 to 250 ppm significantly increased the efficacy of nisin against *L. monocytogenes*. Although a number of studies have assessed nisin efficacy against *L. monocytogenes* on cold-smoked salmon (Nilsson et al., 1997; Neetoo et al., 2008; Ye et al., 2008; Tang et al., 2013; Kang et al., 2014; Chen et al., 2020), none of them investigated the ability of nisin to lower the prevalence of *L. monocytogenes* contamination. In the current study, *L. monocytogenes* was recovered from 33 (61%) of the 54 salmon samples treated with 250 ppm nisin (as compared to 100% of the samples treated with 25 ppm nisin), indicating the potential of highly concentrated nisin treatments to reduce *L. monocytogenes* prevalence. While our statistical analyses indicated that the odds of complete elimination were independent of storage temperature and serotype, the odds of complete elimination were numerically greater (i.e., 2.21 times greater) for salmon stored at 7°C as compared to 4°C. The fact that the difference in odds of complete elimination between storage temperatures was insignificant (*p* value = 0.166) may be due to a relatively small number of samples tested. Assuming the proportions of 250 ppm nisin-treated samples that tested negative (i.e., 8/27 and 13/27 for 4°C and 7°C, respectively) were realistic, a *post hoc* sample size calculation (Dean et al., 2013) indicated we would have needed 214 salmon samples treated with 250 ppm nisin to identify a significant difference (α = 0.05; power = 80%) in the odds of complete elimination achieved at different storage temperatures. This calculation can be used to estimate sample sizes for possible follow-up studies that would specifically assess the ability of high levels of nisin to eliminate *L. monocytogenes* contamination of smoked salmon.

Given the fact that *L. monocytogenes* prevalence was lowered from 100 to 61% in the 250 ppm nisin-treated samples, the theoretical initial log reduction that could be achieved by 250 ppm nisin treatments was estimated to be 3.025. Various studies have investigated the efficacy of nisin to inactivate *L. monocytogenes* on cold-smoked salmon at similar storage temperatures and initial contamination levels (Nilsson et al., 1997; Neetoo et al., 2008). For salmon inoculated with 2.7 log_10_(CFU/cm^2^) *L. monocytogenes* and stored at 4°C, the initial log reduction in *L. monocytogenes* induced by 12.5 ppm and 50 ppm was <1 and 2.7, respectively (Neetoo et al., 2008). In a separate study with an inoculum level of 3.2 log_10_(CFU/g) and a storage temperature of 5°C, the initial log reduction in *L. monocytogenes* following 25 ppm nisin treatment was around 2.2 (Nilsson et al., 1997). This study, for the first time, explored the efficacy of nisin treatment at the maximum allowable level to reduce *L. monocytogenes* on cold-smoked salmon at natural contamination levels, and estimated a theoretical log reduction of 3.025 as well as the possibility of prevalence reduction that could be achieved by this treatment. The results of our study and the previous studies are consistent in suggesting that the nisin efficacy on cold-smoked salmon contaminated with *L. monocytogenes* at natural contamination levels increases in a concentration-dependent manner (higher nisin concentrations lead to higher efficacy). Furthermore, while reduction in prevalence was based on absence of *L. monocytogenes* in 10 g samples, which is lower than both the typical sample size used for testing and the typical package size, our data were also based on (i) an initial inoculation level that could be considered the higher end of natural contamination levels and (ii) *L. monocytogenes* pre-grown under a worst-case scenario (i.e., worst foreseeable conditions that conferred enhanced nisin resistance; Chen et al., 2020). In order to address the impact of different parameters on the complete elimination of *L. monocytogenes*, which could be important in characterizing both public health and recall risks (Pouillot et al., 2007, 2009), the data reported here (and potential new additional data) should be used to conduct risk assessments that model realistic package sizes and include realistic distributions for initial contamination levels.

While our use of low inoculum levels allowed us to identify the potential of treatment with high nisin levels to eliminate *L. monocytogenes*, we also found that nisin efficacy in samples that did not show complete *L. monocytogenes* elimination was overall comparable between low and high *L. monocytogenes* inoculum levels. More specifically, our study here found 0.52, 1.68 and 0.96 log lower *L. monocytogenes* levels on days 1, 15, and 30, respectively, on salmon treated with 25 ppm nisin as compared to the untreated controls, while a previous study conducted in our lab following similar experimental settings (e.g., pre-growth condition, storage temperature, and nisin concentration) but with an inoculum size of 10^6^ CFU/g showed 1.07, 1.06, and 0.57 log lower *L. monocytogenes* levels, also for days 1, 15, and 30, respectively (Chen et al., 2020). Hence, for each day we saw a < 0.7 log difference between the low and high inoculation level experiments. Admittedly, the agreement between the results of this study and our previous study may be due in part to the inclusion of the same set of strains. However, high and low inoculation studies need to be performed with the same set of strains for the comparisons of results to be deemed appropriate since strain variability with respect to nisin susceptibility has been reported by previous studies (Rasch and Knøchel, 1998; Katla et al., 2003; Chen et al., 2020) and observed in this study, as further discussed in sections below. Therefore, our findings suggest that the nisin efficacy determined in challenge studies using high inoculation levels can be properly used for guiding industry practices and incorporated in risk assessments for predicting public health and/or recall risks.

### Nisin efficacy against *Listeria monocytogenes* on cold-smoked salmon appears to not be impacted by storage at mildly abusive temperature (i.e., 7°C)

Not surprisingly, our data confirmed reduced growth of *L. monocytogenes* on salmon stored at 4°C relative to 7°C, consistent with a number of previous studies (Cornu et al., 2006; Hwang, 2007). Importantly, however, we found no difference in the efficacy of nisin (defined as the log reduction in *L. monocytogenes* numbers in nisin-treated salmon samples relative to the untreated controls) at 4 and 7°C, as supported by the observation that the interaction between nisin concentration and storage temperature was not significant. This finding is consistent with a previous challenge study of *L. monocytogenes* on cold-smoked salmon; when inoculated at 2.7 log_10_(CFU/cm^2^) and challenged with nisin at 12.5 or 50 μg/cm^2^, the reduction in *L. monocytogenes* levels on samples stored at 4°C was equal to or lower than on samples stored at 10°C at the end of storage (Neetoo et al., 2008). Different from these findings, an increased nisin sensitivity of *L. monocytogenes* at low temperatures has also been reported. Li et al. (2002) specifically showed that, compared with 30°C, growth of *L. monocytogenes* at 10°C induced an increase in cell membrane fluidity through elevating the percentage of shorter, branched-chain fatty acids and the ratio of anteiso- to iso-configured fatty acids, which was suggested to render the membrane more sensitive to nisin. Increased nisin efficacy against *L. monocytogenes* has also been reported in growth media when temperature was lowered from 12°C to 4°C (Szabo and Cahill, 1998) and in a laboratory-scale cheese model following incubation at 6°C, compared with 14°C and 22°C (Henderson et al., 2020). Many of these studies, however, only identified a reduced nisin efficacy at temperatures typically considered above the typical exposure temperatures of commercially distributed salmon. We thus conclude that nisin appears to maintain the relative added margin of safety at mildly abusive temperatures, which we consider as up to 7°C and possibly up to 10°C, as supported by Neetoo et al. (2008). At abusive temperatures above 10°C one, however, would have to expect a reduced efficacy of nisin. Further studies with larger sample sizes (and *a priori* sample size calculations) may, however, be needed to further clarify the impact of storage temperature on nisin sensitivity.

Biologically, the absence of the impact of storage temperature on nisin efficacy observed here could be attributed to a variety of reasons. Firstly, the temperature difference investigated in this study (4°C versus 7°C) is relatively small and may have limited impact on the fluidity of cell membrane. By comparison, other studies, as detailed above, evaluated nisin sensitivity at wider temperature ranges where differences in membrane fluidity are larger. Secondly, an overall reduction in nisin activity on salmon (relative to other, low-fat matrices, e.g., BHI) may render the difference in nisin efficacy between storage temperatures less apparent. Nisin has been shown to be less effective in inhibiting *L. monocytogenes* in ice cream and fluid milk with higher fat content (Jung et al., 1992; Dean and Zottola, 1996); similarly, binding of nisin molecules to the fat components of salmon could reduce the efficacy of the antimicrobial. Finally, the bacterial culture used in this study was pre-adapted to 0.5 μg/ml benzalkonium chloride, a pre-growth condition that was reported to provide cross-protection for *L. monocytogenes* against the subsequent nisin treatments (Chen et al., 2020), which also could reduce the effect of temperature differences on nisin sensitivity.

### Strain diversity needs to be considered in challenge studies as efficacy of nisin treatments varies depending on serotype, which is affected by antimicrobial concentration and storage temperature

A significant effect of serotype on *L. monocytogenes* levels on salmon was shown for (i) samples treated with 25 ppm nisin and (ii) samples stored at 7°C, as supported by significant two-way interactions between serotype and nisin concentration and between serotype and storage temperature, respectively. Specifically, our data suggests that 1/2b strains may be more susceptible to 25 ppm nisin following incubation at 7°C, as compared to serotypes 1/2a and 4b strains. Similarly, our previous study on salmon conducted with a high inoculation level also demonstrated a higher susceptibility of 1/2b strains to 25 ppm nisin at 7°C, as compared to the serotype 1/2a and 4b strains (Chen et al., 2020). These findings are consistent with a number of studies showing that *L. monocytogenes* from different serotypes can differ in their tolerance to various types of stresses (Lianou et al., 2006; Van Der Veen et al., 2008; Ribeiro and Destro, 2014; Hingston et al., 2017). For instance, serotype 4b strains have been shown to be less susceptible to salt stress (Ribeiro and Destro, 2014), but more susceptible to cold stress (Lianou et al., 2006), compared with strains from serotypes 1/2a and 1/2b. With regard to nisin, different associations between *L. monocytogenes* serotype and nisin susceptibility have been reported. Both serotype 1/2a (Buncic et al., 2001; Szendy et al., 2019) and 4b strains (Ukuku and Shelef, 1997; Henderson et al., 2020) have been reported to be more resistant to nisin, as compared to serotype 1/2b strains, consistent with our results reported here. Some other studies, however, found no effect of serotype on nisin susceptibility (Ferreira and Lund, 1996; Rasch and Knøchel, 1998; Martínez and Rodríguez, 2005). Despite the reported inconsistent results, which could be attributed to the considerable within-serotype strain diversity, the data generated to date suggest that serotype 1/2b strains are in general more susceptible to nisin as compared to strains from serotypes 4b and 1/2a. Hence, our study further emphasizes the importance of using multiple strains encompassing different serovars in challenge studies, which is in line with the suggestions of the “EURL *Lm* Technical Guidance Document on challenge tests and durability studies for assessing shelf-life of ready-to-eat foods related to *L. monocytogenes*” (Bergis et al., 2021), and more specifically stresses that use of solely a serotype 1/2b strain (or strains) may lead to overestimation of the efficacy of nisin treatments. That being the case, the use of one strain from each of the three serotypes in this study by no means represented the entire diversity of *L. monocytogenes* strains. Although hundreds of *L. monocytogenes* strains have been characterized for their natural susceptibility to nisin in culture medium (Rasch and Knøchel, 1998; Katla et al., 2003), we are not aware of similar large-scale studies on cold-smoked salmon. As nisin susceptibility has been reported to differ by matrices (e.g., culture medium vs. salmon), we deliberately selected strains that showed the highest (FSL L3-0051) and lowest (FSL F2-0310) susceptibility to nisin on cold-smoked salmon across different conditions, as suggested by our previous study (Chen et al., 2020). Therefore, the main conclusions of this study regarding nisin efficacy and its variation due to the impact of different factors should be robust and appropriate to support decision making with regard to nisin use on commercial cold-smoked salmon.

## Conclusion

The current study demonstrates the concentration-dependent efficacy of nisin treatments against *L. monocytogenes* at natural contamination levels on cold-smoked salmon and highlights the potential of highly concentrated nisin (250 ppm) to reduce the prevalence of *L. monocytogenes* contamination among the salmon products. Our study also supports that a thorough consideration of inoculum size, storage temperature, strain or serotype diversity, and worst-case scenarios that may confer cross-protection is important when assessing the efficacy of nisin treatments at a given concentration. The information gathered in the current study, such as those regarding odds of complete elimination of *L. monocytogenes* on salmon and *L. monocytogenes* levels on salmon under different combinations of storage temperature and antimicrobial concentration, may also be incorporated into existing and new risk assessment models for a better prediction of the reduction in risks of human listeriosis or food recalls that can be achieved by nisin treatments.

## Data availability statement

The datasets presented in this study can be found in the GitHub online repositories under the direct link: https://github.com/FSL-MQIP/LowInoculation_Listeria_Nisin.git (Name: LowInoculation_Listeria_Nisin).

## Author contributions

RC and JS performed experiments. RC performed statistical analysis. MW and VG-O conceived the study. RC, MW, and VG-O wrote the manuscript. All authors contributed to the article and approved the submitted version.

## Funding

This publication is a product resulting from project R/SHH-18 funded under award NA18OAR4170096 from the National Sea Grant College Program of the US Department of Commerce’s National Oceanic and Atmospheric Administration, to the Research Foundation for State University of New York Sea Grant. The statements, findings, conclusions, views, and recommendations are those of the author(s) and do not necessarily reflect the views of any of those organizations.

## Conflict of interest

The authors declare that the research was conducted in the absence of any commercial or financial relationships that could be construed as a potential conflict of interest.

## Publisher’s note

All claims expressed in this article are solely those of the authors and do not necessarily represent those of their affiliated organizations, or those of the publisher, the editors and the reviewers. Any product that may be evaluated in this article, or claim that may be made by its manufacturer, is not guaranteed or endorsed by the publisher.

## Footnotes

^1^ https://github.com/FSL-MQIP/LowInoculation_Listeria_Nisin.git
