# Supplementary material for: The efficacy of nisin against Listeria monocytogenes on cold-smoked salmon at natural contamination levels is concentration-dependent and varies by serotype
Source: Front Microbiol. 2022 Sep 6;13:930400. doi: 10.3389/fmicb.2022.930400 (PMC9486479; doi:10.3389/fmicb.2022.930400)
Supplement: Supplementary file 1 [file Data_Sheet_1.docx]

**Supplemental Figure 1.** Linear relationship between estimates of *L. monocytogenes* levels on cold-smoked salmon samples obtained using the most probable number technique (log_10_ (MPN/g)) and quantification with direct plating (log_10_ (CFU/g)) based on simple linear regression. The linear equation used for conversion between log_10_ (MPN/g) and log_10_ (CFU/g) is shown in red, and the statistics used to assess the model fit are shown in black. RMSE: Root Mean Square Error.

**Supplemental Table 1.** *L. monocytogenes* levels on cold-smoked salmon samples as represented by log_10_(MPN/g)^a^

| Nisin Concentration (ppm) | Temperature (°C) | Day | Strain | Biological Replicates | | | | | | Average | sd^b^ |
| --- | --- | --- | --- | --- | --- | --- | --- | --- | --- | --- | --- |
|  |  |  |  | 1 | 2 | 3 | 4 | 5 | 6 |  |  |
| 0 | 4 | 1 | FSL F2-0237 | 2.12 | 2.41 | 2.41 | 2.41 | 2.92 | 1.71 | 2.33 | 0.40 |
| 0 | 4 | 1 | FSL F2-0310 | 2.41 | 2.41 | 1.92 | 2.41 | 2.79 | 2.12 | 2.34 | 0.30 |
| 0 | 4 | 1 | FSL L3-0051 | 1.62 | 2.41 | 1.38 | 1.38 | 2.79 | 1.62 | 1.87 | 0.59 |
| 0 | 4 | 15 | FSL F2-0237 | 5.04 | 4.80 | 5.27 | 6.22 | 6.64 | 5.83 | 5.63 | 0.72 |
| 0 | 4 | 15 | FSL F2-0310 | 5.71 | 6.97 | 5.14 | 6.36 | 6.81 | 6.34 | 6.22 | 0.69 |
| 0 | 4 | 15 | FSL L3-0051 | 5.59 | 5.76 | 4.85 | 5.80 | 6.68 | 5.22 | 5.65 | 0.62 |
| 0 | 4 | 30 | FSL F2-0237 | 8.46 | 8.95 | 8.77 | 8.30 | 8.98 | 8.57 | 8.67 | 0.27 |
| 0 | 4 | 30 | FSL F2-0310 | 6.90 | 8.53 | 7.66 | 7.82 | 9.17 | 9.18 | 8.21 | 0.91 |
| 0 | 4 | 30 | FSL L3-0051 | 8.31 | 9.03 | 8.43 | 7.91 | 9.07 | 8.61 | 8.56 | 0.44 |
| 0 | 7 | 1 | FSL F2-0237 | 2.41 | 2.41 | 2.41 | 1.71 | 2.79 | 2.33 | 2.34 | 0.35 |
| 0 | 7 | 1 | FSL F2-0310 | 2.41 | 2.12 | 2.79^c^ | 2.79 | 2.79 | 2.33 | 2.54 | 0.29 |
| 0 | 7 | 1 | FSL L3-0051 | 1.71 | 2.41 | 2.12 | 0.91 | 2.12 | 2.79 | 2.01 | 0.65 |
| 0 | 7 | 15 | FSL F2-0237 | 7.94 | 8.07 | 7.86 | 7.69 | 7.41 | 8.22 | 7.87 | 0.29 |
| 0 | 7 | 15 | FSL F2-0310 | 7.10 | 8.24 | 8.50 | 8.70 | 8.27 | 6.75 | 7.93 | 0.80 |
| 0 | 7 | 15 | FSL L3-0051 | 7.02 | 8.63 | 7.58 | 5.66 | 8.12 | 8.57 | 7.60 | 1.13 |
| 0 | 7 | 30 | FSL F2-0237 | 8.96 | 8.90 | 9.07 | 9.01 | 8.80 | 8.84 | 8.93 | 0.10 |
| 0 | 7 | 30 | FSL F2-0310 | 9.05 | 9.02 | 9.24 | 9.34 | 9.31 | 9.21 | 9.20 | 0.13 |
| 0 | 7 | 30 | FSL L3-0051 | 8.74 | 9.11 | 8.75 | 8.14 | 9.10 | 9.37 | 8.87 | 0.43 |
| 25 | 4 | 1 | FSL F2-0237 | 2.41 | 2.41 | 2.62 | - | - | - | 2.48 | 0.12 |
| 25 | 4 | 1 | FSL F2-0310 | 1.38 | 0.37 | 2.12 | - | - | - | 1.29 | 0.88 |
| 25 | 4 | 1 | FSL L3-0051 | 1.33 | 1.71 | 1.38 | - | - | - | 1.47 | 0.21 |
| 25 | 4 | 15 | FSL F2-0237 | 3.68 | 4.44 | 4.98 | - | - | - | 4.37 | 0.65 |
| 25 | 4 | 15 | FSL F2-0310 | 3.68 | 4.11 | 4.86 | - | - | - | 4.22 | 0.60 |
| 25 | 4 | 15 | FSL L3-0051 | 4.38 | 3.43 | 4.18 | - | - | - | 4.00 | 0.50 |
| 25 | 4 | 30 | FSL F2-0237 | 7.60 | 8.07 | 6.43 | - | - | - | 7.37 | 0.84 |
| 25 | 4 | 30 | FSL F2-0310 | 8.41 | 6.92 | 5.50 | - | - | - | 6.94 | 1.46 |
| 25 | 4 | 30 | FSL L3-0051 | 7.85 | 7.04 | 5.38 | - | - | - | 6.76 | 1.26 |
| 25 | 7 | 1 | FSL F2-0237 | 2.12 | 1.92 | 2.62 | - | - | - | 2.22 | 0.36 |
| 25 | 7 | 1 | FSL F2-0310 | 2.79 | 2.79^c^ | 2.41 | - | - | - | 2.66 | 0.22 |
| 25 | 7 | 1 | FSL L3-0051 | -0.70 | -0.09 | 1.38 | - | - | - | 0.20 | 1.07 |
| 25 | 7 | 15 | FSL F2-0237 | 6.82 | 7.31 | 5.38 | - | - | - | 6.50 | 1.00 |
| 25 | 7 | 15 | FSL F2-0310 | 7.27 | 8.05 | 4.71 | - | - | - | 6.68 | 1.75 |
| 25 | 7 | 15 | FSL L3-0051 | 3.12 | 6.27 | 5.71 | - | - | - | 5.03 | 1.68 |
| 25 | 7 | 30 | FSL F2-0237 | 8.93 | 9.00 | 8.63 | - | - | - | 8.85 | 0.20 |
| 25 | 7 | 30 | FSL F2-0310 | 9.14 | 8.83 | 9.07 | - | - | - | 9.01 | 0.16 |
| 25 | 7 | 30 | FSL L3-0051 | 6.77 | 7.90 | 8.57 | - | - | - | 7.75 | 0.91 |
| 250 | 4 | 1 | FSL F2-0237 | 1.38 | 1.62 | -0.73^d^ | - | - | - | 1.50 | 0.12 |
| 250 | 4 | 1 | FSL F2-0310 | 0.37 | 0.37 | 1.04 | - | - | - | 0.59 | 0.88 |
| 250 | 4 | 1 | FSL L3-0051 | -1.00^d^ | -0.29 | -1.00^d^ | - | - | - | -0.29 | 0.21 |
| 250 | 4 | 15 | FSL F2-0237 | -1.00^d^ | 0.37 | 2.12 | - | - | - | 1.25 | 0.65 |
| 250 | 4 | 15 | FSL F2-0310 | 0.37 | 0.37 | 0.37 | - | - | - | 0.37 | 0.60 |
| 250 | 4 | 15 | FSL L3-0051 | 3.41 | -1.00^d^ | -0.39 | - | - | - | 1.51 | 0.50 |
| 250 | 4 | 30 | FSL F2-0237 | 6.96 | 3.41 | 3.41 | - | - | - | 4.59 | 0.84 |
| 250 | 4 | 30 | FSL F2-0310 | -1.00^d^ | 1.11 | 6.69 | - | - | - | 3.9 | 1.46 |
| 250 | 4 | 30 | FSL L3-0051 | 7.25 | -1.00^d^ | -1.00^d^ | - | - | - | 7.25 | 1.26 |
| 250 | 7 | 1 | FSL F2-0237 | -0.29 | 0.11 | -1.00^d^ | - | - | - | -0.09 | 0.36 |
| 250 | 7 | 1 | FSL F2-0310 | -1.00^d^ | 0.91 | 0.37 | - | - | - | 0.64 | 0.22 |
| 250 | 7 | 1 | FSL L3-0051 | 0.37 | -1.00^d^ | 1.11 | - | - | - | 0.74 | 1.07 |
| 250 | 7 | 15 | FSL F2-0237 | 3.41 | -1.00^d^ | -1.00^d^ | - | - | - | 3.41 | 1.00 |
| 250 | 7 | 15 | FSL F2-0310 | -1.00^d^ | 2.41 | -0.73^d^ | - | - | - | 2.41 | 1.75 |
| 250 | 7 | 15 | FSL L3-0051 | 2.71 | -1.00^d^ | 1.92 | - | - | - | 2.32 | 1.68 |
| 250 | 7 | 30 | FSL F2-0237 | -1.00^d^ | 7.51 | -1.00^d^ | - | - | - | 7.51 | 0.20 |
| 250 | 7 | 30 | FSL F2-0310 | -1.00^d^ | 6.76 | -1.00^d^ | - | - | - | 6.76 | 0.16 |
| 250 | 7 | 30 | FSL L3-0051 | 7.37 | -1.00^d^ | 4.55 | - | - | - | 5.96 | 0.91 |

^a^“-” in the table indicates that no data were collected, as only three biological replicates were performed for samples treated with 25 and 250 ppm nisin.

^b^Standard deviation of log_10_(MPN/g) across biological replicates.

^c^When the *L. monocytogenes* levels on samples exceeded the upper measurement limit of the enumeration method(s); the upper measurement limit is shown instead.

^d^When the *L. monocytogenes* levels on samples were undetectable or below the lower measurement limit of the enumeration method(s), the lower measurement limit is shown instead.
